# The use and methodological reporting of large language models in qualitative research: a scoping review

**DOI:** 10.1186/s12874-026-02913-1

**Published:** 2026-06-16

**Authors:** Christian Kempny, Julian Frings, Paul Rust, Sven Meister, Leonard Fehring

**Affiliations:** 1 Health Services Research, Faculty of Health, School of Medicine, Witten/Herdecke University, Witten, Germany; 2 Health Care Informatics, Faculty of Health, School of Medicine, Witten/Herdecke University, Pferdebachstrasse 11, Witten, 58455 Germany; 3 Department of Gastroenterology, Helios University Hospital Wuppertal, Wuppertal, Germany; 4 Department Healthcare, Fraunhofer Institute for Software and Systems Engineering, Dortmund, Germany

**Keywords:** Large language models, Qualitative research, Artificial intelligence, Scoping review, Reporting guidelines, Thematic analysis, COREQ, Methodological transparency, Human-AI collaboration, Prompt engineering

## Abstract

**Background:**

Large language models (LLMs) are being integrated into qualitative research processes, yet the scope, function, and reporting quality of their use remain poorly understood. Existing reporting guidelines for qualitative research, including for example the Consolidated Criteria for Reporting Qualitative Research (COREQ), provide minimal guidance for documenting LLM use. This scoping review provides an overview of the emerging use of LLMs applications in qualitative research and assesses the associated reporting practices.

**Methods:**

A scoping review was conducted following the PRISMA-ScR guidelines and the Joanna Briggs Institute methodological framework. Five databases (PubMed, CINAHL, PsycINFO, Business Source Premier, and Scopus) were searched for peer-reviewed empirical studies published between January 2020 and May 2025 that employed at least one LLM in a substantive qualitative research stage. The search yielded 5,049 records, of which 4,201 remained after duplicate removal. Studies were screened independently by multiple reviewers, and data were extracted using a standardized template capturing study metadata, methodological characteristics, and comprehensive LLM implementation details.

**Results:**

Seventy-five studies were included. OpenAI GPT models dominated the field, appearing in 93% of studies. LLMs were applied across the full spectrum of qualitative research, with coding assistance (*n* = 43) and theme identification (*n* = 41) as the most common applications. Thematic analysis was the predominant qualitative method (*n* = 38), and content analysis (*n* = 12). Technical reporting was highly inconsistent: only 13 studies reported temperature settings, 12 documented context length, and 4 provided top_p values. Approximately half of studies (45%, *n* = 34) did not specify the deployment configuration (API, web interface, or local), and 75% (*n* = 56) reported no parameter settings at all. While 61% of studies provided complete or partial prompts, 13% reported no prompting details. Agreement rates between LLM and human coders ranged from 36% to 99%, reflecting substantial variation related to task complexity, prompt engineering quality, and validation rigor. Nearly all studies (95%) discussed ethical considerations, and 97% incorporated human verification of AI outputs.

**Discussion:**

LLMs have been adopted across qualitative research workflows, yet critical methodological details are frequently underreported, undermining comparability. The findings highlight an urgent need for dedicated reporting guidelines, such as the COREQ + LLM extension, to ensure that LLM-assisted qualitative research meets standards of transparency, rigor, and interpretive depth. Future research should address the predominance of proprietary models, the limited evidence for non-English contexts, and the need for systematic comparison of models, prompting strategies, and validation approaches.

## Background

Qualitative research enables the systematic investigation of human experiences, meanings, and social processes that are central to understanding human behaviour and social phenomena across a range of disciplines [1, 2]. Through the use of methods such as interviews, focus groups, and document analysis, qualitative studies generate context-sensitive insights that complement quantitative findings and contribute to theory development and practice advancement [3, 4]. To ensure quality and transparency in qualitative research, the use of standardized reporting guidelines has become established practice.

The most prominent framework is the Consolidated Criteria for Reporting Qualitative Research (COREQ) checklist, which has provided structured guidance for reporting interview and focus group studies since 2007 [5]. COREQ has been widely cited (over 35k citations) and adopted, particularly in health research, though its role and appropriateness remain debated within qualitative research communities [6].

Recently, large language models (LLMs) are increasingly being integrated into qualitative research processes. LLMs are generative Artificial Intelligence (AI) systems trained on extensive text corpora that can respond to user inputs (prompts) with fluent natural language outputs [7]. Empirical evidence shows that LLMs are being deployed across various phases of qualitative research: developing interview guides, transcribing and translating qualitative data, supporting coding and theme development, summarizing qualitative datasets, and drafting analytical texts [7–10] .

However, the scope and function of LLM use varies considerably between studies, and precise descriptions of how these tools are methodologically integrated are often lacking. The current reporting practice regarding LLM use in qualitative research is characterized by heterogeneity and inconsistency. In numerous studies, LLMs are merely mentioned, for example, by naming software or a model, without detailed information about the tasks performed, the extent of human involvement, or the procedures for validating and interpreting model outputs. This variability limits the ability to adequately assess methodological rigor, transparency, and reproducibility.

Existing reporting guidelines for qualitative research, including COREQ, were developed before the emergence of LLMs and therefore provide no guidance for documenting their use. While many journals now require generic declarations of AI use, these policies are not tailored to the specific methodological requirements of qualitative research. AI-specific guidelines such as TRIPOD-LLM [11], CONSORT-AI [12], and SPIRIT-AI [13] focus primarily on predictive or interventional study designs and are not tailored to qualitative research using LLMs.

It should be noted that the integration of LLMs into qualitative research is not uncontested. Perspectives within the qualitative research community range from cautious optimism about efficiency gains to fundamental skepticism regarding the compatibility of automated text processing with interpretivist epistemologies and the researcher’s role in meaning-making [14, 15].

Given the rapid and diverse implementation of LLMs in qualitative research, a systematic overview of current applications and reporting practices is urgently needed. Scoping reviews are particularly suited to examining emerging, heterogeneous research fields where concepts, methods, and reporting standards are still evolving [16, 17]. Therefore, this scoping review was conducted to map how LLMs are currently used in qualitative research and how this use is reported in the literature.

The objective of this scoping review is twofold: First, to systematically map existing applications of LLMs use in qualitative research published up to May 2025. Second, to assess the associated reporting practices. Specifically, two central research questions are addressed: (1) How are LLMs currently being applied in qualitative research? (2) What specific reporting practices are used in qualitative studies that employ LLMs?

To answer these questions, this study identifies and categorizes current applications of LLMs as qualitative research instruments across various phases and disciplines of qualitative research. Additionally, it examines how LLM use as a qualitative research instrument is documented in peer-reviewed empirical studies. This assessment includes a thorough examination of the transparency and completeness of methodological documentation, including model specifications, configuration details, parameter settings, prompting strategies, validation approaches, and consideration of limitations and biases.

## Methods

This scoping review was conducted in accordance with the Preferred Reporting Items for Systematic Reviews and Meta-Analyses extension for Scoping Reviews (PRISMA-ScR) guidelines. Following the methodological framework proposed by Arksey and O’Malley [18] and refined by the Joanna Briggs Institute [19], the review comprised four sequential stages: search, screening, data extraction, and synthesis. This scoping review was conducted in accordance with a previously published and pre-specified protocol (COREQ + LLM) [20], which guided the review design, data extraction, and reporting procedures.

### Search strategy

A comprehensive search strategy was developed through iterative refinement and review by an interdisciplinary team of researchers (JF, PR, LF, CK) with expertise in qualitative methodology, LLMs, and research ethics. Five databases were searched through their respective interfaces: PubMed via PubMed, CINAHL via EBSCOhost, PsycINFO via EBSCOhost, Business Source Premier via EBSCOhost, and Scopus via Scopus. The search strategy employed two main concept groups combined with the Boolean operator AND: (1) LLM-related terms including e.g. “large language model,” “generative AI,” “GPT,” “artificial intelligence,” “machine learning,” and (2) qualitative research terms including e.g. “thematic analysis,” “content analysis,” “qualitative research”. The search was limited to peer-reviewed journal articles published in English between January 1, 2020, and May 27, 2025. The complete search strings for each database are provided in the supplementary materials.

### Eligibility criteria

Studies were included if they met the following criteria: (1) peer-reviewed empirical studies; (2) published between January 1, 2020, and May 27, 2025; (3) written in English; and (4) included at least one qualitative method supported by one or multiple LLMs in any stage of the research process, including mixed-methods studies with a qualitative component, beyond manuscript drafting. The restriction to peer-reviewed journal articles was applied to ensure a minimum level of methodological scrutiny and reporting quality, which is particularly relevant when the review itself aims to assess reporting practices. This scoping review did not restrict inclusion to specific disciplines; rather, studies were included based on their use of LLMs within qualitative research processes, regardless of disciplinary context. Studies were excluded if they: (1) employed non-LLM-based software or natural language processing techniques exclusively; (2) were non-peer-reviewed or non-empirical literature including conference abstracts, opinion pieces, dissertations, comments, or editorials; or (3) used LLMs solely for language polishing, grammar correction, or manuscript writing assistance without integrating them into substantive research stages. Transformer-based models without generative capabilities (e.g., BERT, RoBERTa, DistilBERT) were excluded as they represent encoder-only architectures fundamentally distinct from the autoregressive, generative LLMs that are the focus of this review.

### Screening process

The screening process was conducted in four sequential rounds using Rayyan software to facilitate the filtering process and systematically track inclusion and exclusion decisions [21]. First, duplicate records were removed automatically using Rayyan’s deduplication algorithm, which prioritizes matches based on identical digital object identifiers (DOIs) or text similarity of at least 95%. Remaining potential duplicates were checked manually by one screener (CK) to account for metadata inconsistencies. Second, three independent screeners (PR, LF, CK) reviewed titles and abstracts to determine eligibility based on the predefined inclusion and exclusion criteria. Third, four screeners (JF, PR, LF, CK) reviewed full texts of potentially eligible studies. Inclusion required majority agreement. Screeners were instructed to adhere strictly to the predefined inclusion and exclusion criteria throughout all screening stages. Prior to independent screening, the research team collaboratively developed and refined the criteria through iterative discussion of preliminary search results and borderline cases to ensure consistent application. Disagreements during screening were resolved through team discussion until consensus was reached. In accordance with scoping review methodology, no formal quality appraisal of included studies was conducted, as the review aimed to map the breadth of existing evidence rather than assess methodological rigor [16, 19].

### Data extraction

Categories for LLM-specific data elements were derived through an iterative process combining deductive codes informed by the AI reporting frameworks introduced in the Background [11–13] and inductive codes emerging from preliminary data extraction. The resulting extraction categories mapped directly onto the results sections as follows: model type and version (Table 1), qualitative methods (Table 2), purpose of AI usage (Table 3), parameter settings (Table 4), deployment configuration (Table 5), prompting strategies (Table 6), ethical considerations (Table 7), and human-AI interaction patterns (Table 8).

**Table 1:** AI model families and specific versions used in the included studies

AI Tool Family	Frequency	Specific Versions
OpenAI GPT Models	70 studies [7, 8, 10, 17, 22–87]	GPT-3.5-turbo, GPT-4, GPT-4-Turbo, GPT-4o, GPT-turbo-0125
Anthropic Claude	6 studies [7, 31, 40, 53, 64, 65]	Claude 2, Claude 2.0, Claude 3.5 Sonnet, Claude-instant-100 K
Meta LLaMA	5 studies [7, 65, 88–90]	LLaMA-2-70B-Instruct, LLAMA3 70B, LLaMA
Google Models	6 studies [7, 23, 51, 65, 67, 68]	Google Bard, Gemini 1.5 Pro, Gemini 2.0 Advanced
Other Models	9 studies [7, 32, 51, 52, 59, 65, 69, 91, 92]	Mixtral 7 × 8b, DeepSeek-V3, DiVoMiner, RoB-ELoC
Not mentioned	1 study [93]	--

Categories are not mutually exclusive; individual studies may contribute to multiple categories

**Table 2:** Qualitative methods and example applications in included studies

Qualitative Method	Number of Studies	Example Applications
Thematic Analysis	38 [7, 10, 22–25, 27, 28, 32, 33, 35, 41, 42, 47, 48, 51, 52, 55, 57, 58, 63, 64, 67–72, 76–82, 84, 89, 93]	Coding transcripts, generating themes, preprocessing quotes
Content Analysis	12 studies [8, 40, 44, 46, 49, 60, 73, 74, 83, 86, 91, 92]	Identifying markers, detecting adverse events, analyzing social media
Document Analysis	11 studies [31, 36, 39, 56, 61, 65, 66, 75, 85, 87, 91]	Policy analysis, media analysis, webpage analysis
Grounded Theory	6 studies [26, 34, 38, 50, 71, 91]	Open coding, axial coding, selective coding
Further Methods	11 studies [29, 30, 43, 45, 53, 54, 59, 62, 72, 86, 90]	Psychiatric Interview Analysis, Situational Analysis, Discourse Analysis, Autoethnography, Narrative Analysis

Categories are not mutually exclusive; individual studies may contribute to multiple categories

**Table 3:** Purpose categories and corresponding AI applications in included studies

Purpose Category	Studies	Description
Coding assistance	43 studies [8, 22, 26, 29–34, 37–40, 42, 44, 49, 53, 54, 56–58, 60, 61, 63, 65, 71, 74–77, 81–83, 85–93]	Generating codes from qualitative data
Theme identification	41 studies [7, 10, 22–25, 29, 32–36, 40–42, 44, 46–48, 50–52, 55, 57, 58, 63, 66–72, 77–82, 84]	Identify and summarizing themes
Efficiency improvement	15 [7, 8, 25–27, 41, 42, 44, 55, 57, 58, 76, 79, 82, 84]	Reducing time required for analysis
Human-AI comparison	45 studies [7, 8, 10, 22–26, 31, 34–39, 41–43, 46, 48, 50, 53, 54, 56, 58, 62–66, 71, 73–75, 79, 81–89, 93]	Evaluating LLM performance against human coders
Second coder/triangulation	6 studies [25, 60, 67, 68, 77, 80]	Using LLMs for investigator triangulation
Sentiment analysis	7 studies [36, 47, 51, 53, 73, 86, 92]	Assessing emotional tone of text
Data preprocessing	1 study [58]	Cleaning and preparing qualitative data

Categories are not mutually exclusive; individual studies may contribute to multiple categories

**Table 4:** Reported AI model parameters and common settings

Parameter	Studies Reporting	Common Settings
Temperature	13 studies [10, 34, 37, 39, 54, 60, 75, 82, 83, 85–87, 89]	0, 0.2, 0.7, 1.0, 1.31
Context length/tokens	12 studies [10, 25, 26, 31, 46, 58, 64, 79, 83, 86–88]	4,097 tokens, 8,192 tokens, 16,384 tokens, 100 tokens
Top_p	4 studies [83, 85, 87, 89]	0.14, 0.9, 1
Not stated	56 studies [7, 8, 22–24, 27–30, 32, 33, 35, 36, 38, 40–47, 49–53, 55–57, 59, 61–63, 65, 68–74, 76–78, 80, 81, 84, 90, 92, 93]	No parameter settings reported

Categories are not mutually exclusive; individual studies may contribute to multiple categories

**Table 5:** LLM deployment configurations and examples

Configuration	Number of Studies	Examples
API deployment /Web Interface	38 [7, 10, 22, 25, 26, 28, 30, 32–34, 37, 39, 43–45, 47, 53–58, 62–68, 71, 73, 79, 80, 82–87]	OpenAI API
Local deployment	5 [32, 46, 65, 88, 89]	LLaMA on local server, LLAMA3 server-installed, Mixtral locally
Not stated	34 [8, 23, 24, 26, 31, 32, 34, 36–38, 40–42, 44, 46, 48, 51, 52, 58, 59, 61, 64, 65, 70, 72, 74, 76, 77, 80, 81, 87, 91–93]	No deployment information provided

Categories are not mutually exclusive; individual studies may contribute to multiple categories

**Table 6:** Prompting strategies used in included studies

Strategy	Studies Using	Description
Role assignment	20 studies [22, 24, 26, 31, 34, 37, 38, 42, 49, 50, 52, 54, 59, 63, 66, 71, 75, 82, 83, 88]	Instructing LLMs to act as a researcher
Step-by-step instructions	35 studies [8, 22, 24–26, 31, 34, 36–38, 42, 46, 48, 49, 51, 53, 54, 58, 61–63, 66–68, 71, 73–75, 79, 82–84, 87, 88]	Chain-of-thought prompting
Few-shot learning	10 studies [23, 30, 34, 43, 49, 65, 75, 82, 83, 90]	Providing examples in prompts
Zero-shot	10 studies [7, 23, 38, 49, 65, 67, 74, 75, 78, 79]	No examples provided
Iterative refinement	49 studies [7, 8, 10, 22, 23, 25, 26, 29, 32–36, 39, 40, 44, 46–50, 52–62, 67–76, 79, 81, 84, 86, 89, 91]	Multiple rounds of prompting
Structured output format	55 studies [7, 8, 10, 25–28, 31–42, 44, 45, 47–59, 62–65, 68, 69, 72–78, 80, 82–85, 87, 91, 92]	Specifying JSON, tables, or lists

Categories are not mutually exclusive; individual studies may contribute to multiple categories

**Table 7:** Ethical topics and key concerns reported in included studies

Ethical Topic	Studies Addressing	Key Concerns
LLM limitations/caveats	49 studies [7, 10, 22, 23, 25–28, 31, 32, 34–37, 39–41, 43, 45, 48, 53–59, 61–67, 69, 71, 73, 75, 76, 79, 81–87, 89, 93]	Hallucinations, context limitations, reproducibility issues
Bias concerns	51 studies [7, 8, 22, 23, 25, 26, 31–37, 40–43, 45, 46, 48–50, 52–55, 57, 59, 62, 63, 65–67, 69, 71, 73, 75, 76, 78–82, 84–87, 89, 91, 93]	Training data bias, cultural bias
Data privacy	50 studies [7, 8, 10, 23–27, 29, 30, 32–35, 37, 38, 41–44, 46–48, 50–52, 55, 57–59, 62, 66–69, 71, 74, 75, 77, 80–85, 87–89, 91, 92]	Confidentiality
Human oversight necessity	73 studies [7, 8, 22–28, 30–35, 37–40, 59, 61–69, 71, 73–76, 78–81, 84–89, 92, 93]	Need for manual review
Not addressed	4 studies [60, 70, 72, 90]	No ethical discussion

Categories are not mutually exclusive; individual studies may contribute to multiple categories

**Table 8:** Human–LLM interaction patterns in included studies

Interaction Pattern	Studies	Description
Parallel analysis comparison	37 studies [23–26, 28, 29, 31, 33–35, 37, 41, 46–48, 50, 54, 56, 57, 59, 60, 62–64, 66, 67, 71, 72, 74, 76, 79, 81–84, 86, 89]	Side-by-side human and LLM analysis
LLM as assistant/second coder	43 studies [8, 10, 25–27, 29–38, 44, 45, 47–49, 51, 52, 54, 55, 57, 58, 60, 62, 63, 65, 67, 70–74, 78, 79, 81, 83, 85, 87, 88]	LLM supporting human-led analysis
Hybrid/collaborative approach	37 studies [7, 8, 22, 24–35, 39, 40, 42–45, 47–49, 51, 52, 54, 57–63, 65, 88, 93]	Integrated human-LLM workflow

Categories are not mutually exclusive; individual studies may contribute to multiple categories

Data extraction was performed by multiple extractors working in parallel using a standardized extraction template developed collaboratively by the research team. The extractors independently conducted an initial round of data extraction on a pilot sample of 10 studies to test and refine the template, followed by joint discussions to determine final categorization of extracted data elements and ensure consistent application. The template captured: (1) study metadata including authors, year of publication, and country of origin; (2) methodological characteristics including qualitative methods employed and study design; and (3) comprehensive LLM implementation details. The final charting framework covered the following domains: (1) characteristics of the LLM models used (type and version), (2) qualitative research methods applied, (3) purposes and stages of LLM integration, (4) technical configuration and parameter settings, (5) deployment characteristics, (6) prompting strategies, (7) ethical considerations, and (8) patterns of human–AI interaction. The results are presented according to the predefined data charting domains, reflecting key aspects of LLM use and reporting practices identified during data extraction.

### Data aggregation and synthesis

The extracted data were aggregated and synthesized to provide a comprehensive overview of LLM applications and associated reporting practices in qualitative research. Synthesis was conducted using a descriptive, narrative approach, in which extracted variables were grouped into thematic categories and iteratively compared across studies to identify patterns, consistencies, and variations. No formal quality assessment of included studies was conducted, consistent with scoping review methodology which emphasizes mapping the literature rather than evaluating study quality. Similarly, no publication bias analyses or sensitivity analyses were planned or conducted. The synthesized results were prepared for presentation in both narrative and tabular formats.

**Table 9:** Overview of included studies and applied AI tools

Study	Qualitative Method	Primary AI Tool	Country	Study Aim / Purpose of LLM use
Alexander et al., 2025 [46]	Facilitated discussion groups with thematic analysis	ChatGPT 4.0	United States	Compare LLM-driven thematic analysis with human-coded analysis to test equivalence with the gold-standard method.
Arillotta et al., 2024 [52]	Thematic analysis; phenomenological media analysis	Numerous.ai; ChatGPT 3.5	United Kingdom	Assist thematic analysis to identify themes and biases related to GLP-1 RAs and substance use/behavioural addictions.
Balt et al., 2025 [88]	Semi-structured interviews; deductive coding and summarisation	LLAMA3 70B instruct	Netherlands	Evaluate LLM feasibility for deductively coding and summarising psychosocial autopsy interview data.
Bennis et al., 2025 [7]	Thematic analysis	Nine LLMs incl. ChatGPT o1-Pro, Claude 3.5 Sonnet, Gemini 2.0, DeepSeek V3, Llama 3.1	Morocco	Assess efficacy of nine LLMs in thematic analysis of the psychosocial impact of cutaneous leishmaniasis and compare with manual methods.
Bijker et al., 2024 [8]	Content analysis (inductive & deductive); document analysis of forum posts	ChatGPT (GPT-3.5-turbo)	New Zealand	Explore ChatGPT’s utility for qualitative content analysis across inductive and deductive approaches, reducing human workload.
Booyse et al., 2024 [93]	Qualitative interviews; AI-supported thematic coding	ATLAS.ti with AI (model not specified)	South Africa	Automate transcript coding and compare with human-generated coding for accuracy and consistency.
Borgström et al., 2025 [45]	Computational text analysis of parliamentary debates	ChatGPT (16 K); OpenAI text-embedding-3-small	Sweden	Apply LLM-supported computational text analysis to 45 years of parliamentary debates to scale discourse analysis.
Bouton et al., 2024 [70]	Thematic analysis	ChatGPT	United States	Thematic analysis of qualitative data.
Brondani et al., 2024 [35]	Thematic analysis	ChatGPT	Canada	Test whether instructors can differentiate ChatGPT-generated reflections from student work and compare thematic analyses with qualitative researchers.
Carvalho et al., 2024 [23]	Thematic analysis	ChatGPT 3.5 and 4; Google Gemini	Canada	Assess LLM ability to identify and summarise topics in open-ended responses as a preliminary, supervised tool.
Castellanos et al., 2025 [66]	Document analysis; topic modelling with LLM-assisted interpretation	GPT-3.5; GPT-4	Canada	Compare human interpretation with LLM-derived labels in the interpretation stage of topic modelling.
Castellanos-Reyes et al., 2025 [83]	Content analysis (student text data)	GPT-turbo-0125	United States	Automate content analysis of student text based on PIM indicators and assess reliability versus human analysis.
Cevik et al., 2025 [68]	Thematic analysis (survey feedback)	ChatGPT; NoteBookLM; Custom-GPT	UAE	Conduct inductive thematic analysis and triangulation of course feedback and build a specialised GPT for qualitative work.
Curry et al., 2024 [56]	Discourse analysis (corpus approaches)	ChatGPT-4	United Kingdom	Investigate ChatGPT’s affordances for automated qualitative analyses within corpus approaches to discourse studies.
De Paoli et al., 2024 [10]	Inductive thematic analysis of semi-structured interviews	GPT-3.5-Turbo	Scotland	Explore whether LLMs can perform inductive thematic analysis of semi-structured interviews and assess validity against prior analyses.
Deiner et al., 2024 [85]	Document analysis (tweets); AI-supported probabilistic assessment	GPT-3.5; GPT-4	United States	Assess whether tweets about conjunctivitis indicate regional outbreaks and assign outbreak probabilities to posts.
Deiner et al., 2024 [64]	Thematic analysis; topic model selection (social media)	GPT-4–32 K; Claude-instant-100 K; Claude-2–100 K	United States	Assess feasibility of LLMs for topic selection and inductive thematic analysis of large health-related social media datasets.
Domenach et al., 2024 [60]	Content analysis (interview transcripts)	GPT-3.5-Turbo	France	Triangulate manual content analysis to identify psychosocial and contextual markers physicians use to personalise care.
Dos Anjos et al., 2024 [31]	Interviews; prompt-based qualitative analysis	ChatGPT 4.0; Claude 2.0	Brazil	Evaluate LLMs for qualitative research in science education, including analysis of students’ conceptual understanding.
Falissard et al., 2025 [80]	Interviews; thematic analysis	ChatGPT-4	France	Triangulate human thematization and provide a more neutral corpus analysis in a child and adolescent psychiatry study.
Fan et al., 2024 [39]	Content analysis (human–LLM coordinated)	GPT-3.5-turbo-1106; GPT-4o	Hong Kong	Collaborate with human coders to measure complex latent concepts (e.g., deliberativeness) by refining codebooks and prompts.
Fanning et al., 2024 [61]	Document analysis; content analysis of webpages	ChatGPT 3.5 and 4.0	United States	Automate content analysis and improve readability of plastic surgery webpages on breast implant size selection.
Fennig et al., 2025 [36]	Document analysis of online forum posts	ChatGPT-4 Turbo; GPT-4o	Israel	Identify recurring themes and sentiment in epilepsy forum posts and classify cross-posting to depression/suicide subreddits.
Fuller et al., 2024 [55]	Thematic analysis (course evaluations)	ChatGPT	United States	Analyse course evaluation comments and compare agreement between instructor-identified and AI-identified themes.
Gandy et al., 2025 [73]	Content analysis (sentiment)	ChatGPT 4.0	United States	Evaluate ChatGPT’s performance in sentiment analysis of YouTube comments against VADER, T2D, and LIWC.
Hara et al., 2025 [47]	Semi-structured interviews; thematic and emotional analysis	ChatGPT-4o	Japan	Analyse interview transcripts of operating-room nurses for thematic, emotional, and subjectivity content.
He et al., 2021 [91]	Content analysis; grounded theory (tweets)	LSTM network	United States	Identify and categorise tweets, classify opinions on mask wearing, and understand rationales behind anti-mask sentiment.
Hitch et al., 2024 [33]	Reflexive thematic analysis; document analysis	ChatGPT-4	Australia	Augment reflexive thematic analysis by generating summaries, codes, patterns, and themes in health research.
Jalali et al., 2024 [62]	Interview data analysis (causal loop diagrams)	ChatGPT (GPT-4)	United States	Replicate analysis of interview data to create a causal loop diagram and compare results with the original analysis.
Jiang et al., 2025 [79]	Thematic analysis of interview transcripts	GPT-3 (Davinci)	United States	Explore feasibility and comparability of GPT-3 for thematic analysis in equity-focused education research.
Kasperiuniene et al., 2024 [29]	Situational analysis (document analysis of TED Talks)	ChatGPT-4	Lithuania	Integrate ChatGPT-4 into situational analysis of TED Talks and explore AI roles as co-analyst, consultant, and trainer.
Kon et al., 2025 [84]	In-depth interviews	ChatGPT 3.5 and 4.0	Singapore	Compare themes generated by ChatGPT with traditional human analysis of in-depth interviews and assess feasibility for rapid preliminary analysis.
Kondo et al., 2024 [24]	Thematic analysis of interview data	ChatGPT (GPT-4)	Japan	Evaluate ChatGPT’s applicability in thematic analysis for medical qualitative research in deductive, inductive, and abductive approaches.
Koželj et al., 2025 [40]	Semi-structured interviews; LLM-assisted content analysis	Claude 3.5 Sonnet; GPT-4	Slovenia	Assist qualitative content analysis of interview transcripts using iterative prompting with human oversight.
Leas et al., 2024 [87]	Document analysis (social media posts)	ChatGPT (gpt-3.5-turbo-0613)	United States	Identify adverse events in social media posts and compare ChatGPT’s performance with human annotators.
Lee et al., 2024 [58]	Thematic analysis; transcript generation	ChatGPT 3.5 and 4.0	Canada	Explore ChatGPT’s utility in direct coding, theme generation, quote preprocessing, and training transcript generation.
Lee et al., 2025 [27]	Thematic analysis of open-ended responses	GPT-4o	Australia	Identify presence/absence of themes and subthemes in participants’ responses to manage large qualitative datasets efficiently.
Li et al., 2024 [41]	Semi-structured interviews; qualitative description	GPT-4 (private instance, Versa)	United States	Evaluate GPT-4’s effectiveness in analysing interviews on adult-acquired buried penis versus human researchers.
López-Pérez et al., 2025 [53]	Narrative analysis (open-ended narratives)	ChatGPT-4; Claude 2	United Kingdom	Evaluate feasibility of categorising interpersonal emotion regulation strategies and provide an alternative measurement approach.
Maltby et al., 2024[78]	Thematic analysis	ChatGPT-4	United Kingdom	Use ChatGPT-4 to identify initial themes and create superordinate themes in a multidisciplinary analysis.
Mannstadt et al., 2024 [25]	Semi-structured interviews; focus groups; thematic analysis	ChatGPT-4	United States	Support investigator triangulation in mixed-methods research via thematic analysis and survey generation.
Mathis et al., 2024 [89]	Thematic analysis of semi-structured interviews	LLaMA-2-70B-Instruct (local)	United States	Compare locally hosted LLMs with traditional human thematic analysis on semi-structured interviews in a psychiatric setting.
Mayring et al., 2025 [74]	Qualitative content analysis with inductive category formation (interviews)	ChatGPT 3.5 and 4	Germany	Examine ChatGPT’s contribution to qualitative content analysis of interview texts and its suitability for specific methods.
Meinert et al., 2025 [77]	Focus groups; thematic analysis	ChatGPT 3.5	United Kingdom	Enhance thematic analysis by providing an additional perspective on ATLAS.ti-generated codes.
Mizumoto et al., 2025 [65]	Document analysis with LLM-supported classification	GPT-4o; GPT-o1; GPT-o3-mini; Llama 3.3-70B; Gemini 2.0 Flash; Claude 3.5 Sonnet; DeepSeek-V3	Japan	Classify open-ended learner responses into self-regulated learning processes and evaluate accuracy and methodological implications.
Morse et al., 2025 [63]	Semi-structured interviews; grounded-theory-based thematic content analysis	ChatGPT-3.5	United States	Assess ChatGPT’s ability to generate interview questions, create a codebook, and perform thematic content analysis.
Mumba et al., 2025 [51]	Focus group discussions; hybrid thematic analysis	Gemini 1.5; ChatGPT 4.0; AILYZE	Zambia	Identify patterns, explore theme relationships, and perform sentiment analysis on focus-group discussion transcripts.
Panke et al., 2025 [59]	Digital autoethnography	ChatGPT; Pictory.ai; HeyGen; others	Germany	Document generative AI as both research partner and subject across educational research, instructional design, and teaching.
Perkins et al., 2024 [28]	Inductive thematic analysis; document analysis	ChatGPT (GPT-4)	Turkey / United Kingdom	Support qualitative data analysis by integrating GenAI with traditional manual methods for deeper interpretation.
Perkins et al., 2024 [81]	Inductive thematic analysis; document analysis	ChatGPT Plus (GPT-4)	Vietnam / Singapore	Conduct inductive thematic analysis of publisher policies on Generative AI and support theme development.
Player et al., 2025 [82]	Inductive thematic analysis via DECOTA (structural topic modelling + fine-tuned LLMs)	GPT-3.5 Turbo 0613	United Kingdom	Use LLMs to interpret NLP outputs and automatically identify codes and themes from free-text data.
Prescott et al., 2024 [42]	Inductive and deductive thematic analysis (SMS prompts)	ChatGPT-3.5; Bard (PaLM 2)	Canada	Evaluate LLM consistency and reliability in thematic analyses compared with human coders in digital health interventions.
Prinzing et al., 2025 [37]	Document analysis (spirituality coding)	GPT-3.5; GPT-4	United States	Code texts for spirituality and compare performance with human raters regarding inter-rater reliability and validity.
Qiao et al., 2025 [57]	Thematic analysis of semi-structured interviews	ChatGPT-4	United States	Automate inductive coding of interview transcripts to extract themes from qualitative interview data.
Randerson et al., 2025 [54]	Discourse Network Analysis of news articles and interviews	GPT-4	New Zealand	Build a codebook for discourse network analysis of alcohol policy reform and evaluate alignment with human coders.
Sakaguchi et al., 2025 [50]	Semi-structured interviews; grounded theory	ChatGPT-4	Japan	Evaluate feasibility, strengths, and limitations of ChatGPT-4 in analysing Japanese interview data, including cultural nuance.
Shlobin et al., 2024 [69]	Layered ChatGPT-based thematic analysis; Pareto analysis	ChatGPT (GPT-3.5); Bing Chat (GPT-4); Bard; others	United States	Synthesise themes and identify opportunities and considerations for integrating AI into global neurosurgery.
Slotnick et al., 2025 [48]	Reflective thematic analysis of open-ended responses	Google Bard; ChatGPT-3.5	United States	Enhance qualitative research in higher education assessment through AI-assisted theme extraction and analysis.
Smirnov et al., 2025 [49]	Directed and conventional content analysis; narrative analysis	GPT-4	Spain	Explore LLM potential in qualitative psychological research for exploratory studies, narrative analysis, and text evaluation.
Smith et al., 2025 [90]	BERTopic modelling; ideological scoring	LLaMA model	United States	Generate labels for latent topics and estimate ideological scores of YouTube videos.
So et al., 2024 [30]	Semi-structured psychiatric interviews	GPT-4 Turbo; fine-tuned GPT-3.5 Turbo	South Korea	Identify sections of psychiatric interviews indicating symptoms and summarise stressors and symptoms to support clinical workflows.
Stage et al., 2025 [22]	Thematic analysis of open-ended surveys	ChatGPT	United States	Analyse qualitative health services feedback from LGBTQ+ patients alongside human coding to inform future interventions.
Stavropoulos et al., 2024 [75]	Document analysis (classification of psychological constructs)	GPT-4; GPT-3.5; RoB-ELoC	Canada	Classify constructs such as intellectual humility and perspective-taking and test LLM-assisted coding pipelines.
Sun et al., 2025 [43]	Interviews (with AI-generated questions)	ChatGPT-3.5	China	Evaluate ChatGPT as a support tool for investigative interviews with children regarding question formulation and information elicitation.
Tabone et al., 2023 [86]	Sentiment analysis, summarisation, think-aloud analysis	ChatGPT (gpt-3.5-turbo)	United Kingdom	Evaluate ChatGPT as a tool for qualitative data analysis in HCI research (questionnaires, interviews, think-aloud data).
Wachinger et al., 2025 [71]	Semi-structured interviews; thematic analysis; grounded theory; reflexive TA	ChatGPT (GPT-3.5)	Germany, USA, South Africa	Compare ChatGPT’s performance in qualitative data analysis with a human researcher and assess implications for practice.
Wang et al., 2025 [67]	Thematic analysis of semi-structured interviews	GPT-4; Gemini 1.5 Pro	United States	Provide supplementary assistance in thematic analysis of semi-structured interviews using Braun and Clarke’s six-phase framework.
Winters et al., 2025 [72]	Phenomenological analysis; ChatGPT-enhanced thematic and meta-analysis (interviews)	ChatGPT	New Zealand	Analyse participant excerpts to identify themes related to provider meaning and emotions and identify potential human bias.
Wosny et al., 2024 [32]	Thematic analysis of interviews	gpt-3.5-turbo; Mixtral 7 × 8b	Switzerland	Support semi-automated interpretation of qualitative interview data in healthcare via inductive and deductive thematic analysis.
Wu et al., 2025 [92]	AI-aided content analysis; semantic network analysis	DiVoMiner	China	Analyse suicide notes via word frequency, semantic networks, and sentiment analysis to understand content and emotional tone.
Yang et al., 2025 [34]	Interviews; inductive coding; grounded theory; theory-driven thematic analysis	GPT-4 Turbo	United States	Test LLM application in qualitative analysis, assess reliability/validity, and evaluate capacity for theory-driven TA.
Yue et al., 2025 [26]	Grounded theory with semi-structured interviews	ChatGPT 4-Turbo	China	Evaluate ChatGPT’s performance in coding and grounded-theory analysis compared with manual methods and provide a tutorial.
Zhang et al., 2024 [44]	Focus groups; thematic analysis	ChatGPT 3.5	Malaysia	Summarise focus-group transcripts, identify key excerpts, generate key phrases, and compare with NVivo-based analysis.
Zhang et al., 2024 [76]	Semi-structured interviews; content analysis	ChatGPT-4	United States	Assist coding and analysis of interview data to improve efficiency, consistency, and transparency of qualitative analysis.
Zhou et al., 2024 [38]	Grounded theory; document analysis	ChatGPT-4	Canada, China	Apply a grounded-theory approach to risk analysis of electric power system blackouts and evaluate performance against manual methods.

## Result

**Fig. 1:**
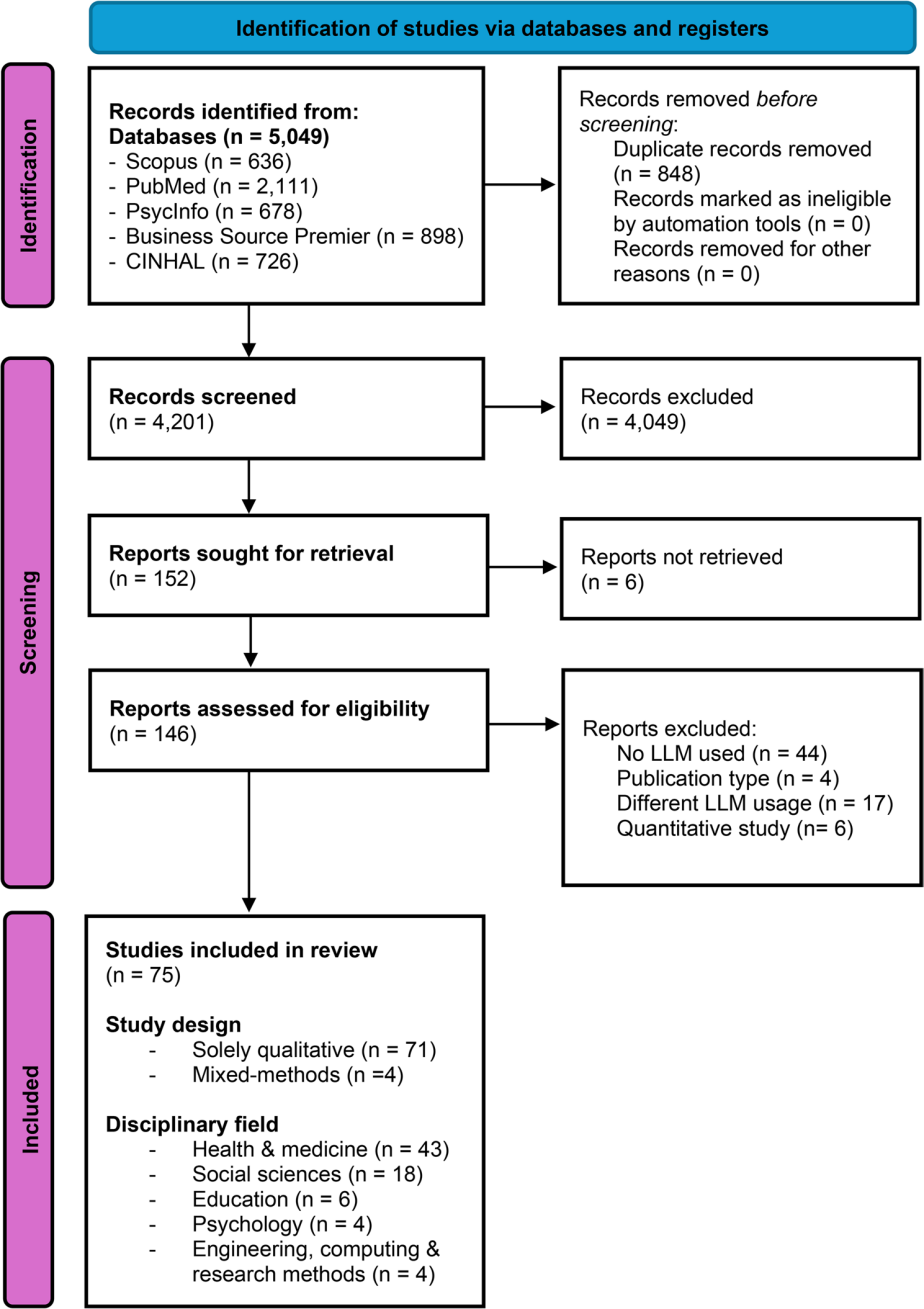
PRISMA 2020 flow diagram

The 75 included studies spanned five disciplinary fields, with health and medicine most strongly represented (*n* = 43, 57%), followed by social sciences, humanities and communication (*n* = 18, 24%), education (*n* = 6, 8%), psychology and behavioural sciences (*n* = 4, 5%), and engineering, computing and research methods (*n* = 4, 5%). Seventy-one studies used solely qualitative designs, while four employed mixed-methods design. The studies spanned multiple countries, methodological approaches, and AI tools. The included studies represented research from at least 20 different countries based on author affiliations, with the United States being the most frequently represented (*n* = 26, 35%), followed by the United Kingdom (*n* = 9, 12%), Japan and China (*n* = 4 each, 5%), Germany and New Zealand (*n* = 3 each, 4%), and Australia and France (*n* = 2 each, 3%) (Fig. 1).

Detailed documentation of LLM utilization included: (1) the specific LLM model type and version (such as GPT-4, GPT-3.5, Claude, Llama, or Gemini) (see Tables 9 and 1 ); (2) model configuration details (application programming interface (API) access, web-based interface, or local deployment) (see Table 5); (3) parameter settings including temperature, top-p, context length, and maximum tokens (see Table 4); (4) purpose and stage of integration in the research process (such as coding, theme development, transcription, translation, or analysis) (see Table 3); (5) prompting strategies including whether prompts were provided, the approach used (zero-shot, few-shot, iterative, multi-stage, or structured), and examples when available (see Table 6); (6) nature of human-AI interaction including manual review, iterative refinement, or prompt adjustment (see Table 8); and (7) reported outcomes or findings specifically attributed to LLM use.

### AI tools and models used

The studies employed a diverse range of AI tools and LLM models for qualitative research applications. OpenAI’s GPT models were most frequently used, appearing in 93% of studies. ChatGPT-4 and its variants (including GPT-4-Turbo and GPT-4o) were the most used, followed by GPT-3.5-turbo. Several studies employed multiple models for comparison purposes. Open-source models were notably used less frequently than commercial offerings. Some authors specifically utilized locally-hosted open-source LLMs to address data privacy concerns with protected health information (see Table 1).

AI applications spanned the full spectrum of qualitative research methods. Thematic analysis was the predominant method, with AI used across multiple phases including initial coding, theme generation, and theme refinement (see Table 2). Bijker et al. [8] describe the use of ChatGPT to assist in each phase of qualitative content analysis, including data identification, coding scheme development, and application of coding schemes. Similarly, Qiao et al. [57] reported that GenAI was used to identify themes from interview transcripts with reported agreement rates of over 80%.

For grounded theory applications, Zhou et al. [38] proposed a novel approach guiding ChatGPT through qualitative data analysis processes, including open coding, axial coding, selective coding, and data saturation testing with the authors reporting results they considered comparable to those of human analysts. Yue et al. [26] provided a step-by-step tutorial on applying ChatGPT 4-Turbo to grounded theory within qualitative research workflows.

### Purposes of LLM usage in qualitative research

The purposes for which LLMs were employed varied considerably across studies. Several studies reported reductions in analysis time within their respective study contexts when applying LLMs (see Table 3). For example, Kon et al. [84] reported a reduction in analysis time from 240 min to approximately 11–12 min per transcript, while Player et al. [82] and Prescott et al. [42] reported similar patterns in terms of time and cost efficiency.

LLMs were also used as a tool for enhancing research rigor through triangulation. Mannstadt et al. [25] proposed integrating LLMs and human investigators as a supplementary tool for investigator triangulation in mixed-methods research. Falissard et al. [80] employed double triangulation using human coders and LLMs in their thematic analysis .

### Technical configuration and parameter reporting

A substantial proportion of studies (75%) did not report specific parameter settings (see Table 10 for definitions of technical terms) for the LLMs used (see Table 4). When temperature (a parameter controlling randomness in model outputs, where lower values produce more deterministic responses and higher values increase variation) was reported, settings varied from 0 (for reproducibility) to 1.31 (for creativity). Tabone et al. explicitly set temperature to 0 for accurate, reproducible results, while Mathis et al. used a temperature of 1.31 along with top_p of 0.14, repetition_penalty of 1.17, and top_k of 49 [89].

**Table 10:** Glossary of key technical terms related to LLM use in qualitative research

Term	Definition
API (Application Programming Interface)	A programmatic way to access and interact with an LLM, typically used for automated or large-scale analysis workflows.
Commercial models	Proprietary models provided by companies (e.g., OpenAI GPT), typically accessed via API or web interface.
Context length	The maximum amount of text (input and output combined) that a model can process at once, typically measured in tokens.
Few-shot prompting	Providing a small number of examples within the prompt to guide the model’s response.
Iterative prompting	Refining model outputs through multiple rounds of prompting and adjustment.
Local deployment	Running an LLM on local hardware or private servers, often used to ensure data privacy.
Open-source models	Models whose architecture and/or weights are publicly available and can be used, modified, and hosted independently (e.g., LLaMA).
Structured prompting	Using predefined formats (e.g., tables, JSON, lists) to guide model outputs.
Temperature	A parameter controlling the randomness of model outputs. Lower values produce more deterministic and consistent responses, while higher values increase variability and creativity.
Token	A token is a unit of text used by a language model, such as a word, part of a word, or punctuation. The number of tokens determines how much text the model can process or generate at once.
Top-p (nucleus sampling)	A parameter that limits the model’s output to a subset of probable next tokens whose cumulative probability exceeds a specified threshold, influencing diversity of responses.
Web interface	A browser-based interface (e.g., ChatGPT) where users interact with the model manually.
Zero-shot prompting	Providing a task to the model without any examples.

45% of the studies did not specify whether LLMs were accessed via API, web interface, or local deployment (see Table 5). It is likely that many of these studies without stated deployment methods used web interfaces, as this represents the most accessible entry point for researchers without technical expertise in API integration or local model deployment. Among those that did specify, API deployment was most common. Local deployment was specifically chosen by studies handling sensitive data, such as Mathis et al. [89] who used a locally-hosted LLM on an Ubuntu Linux server with dual Nvidia RTX 3090 GPUs to process protected health information .

Prompting strategies varied widely across studies, ranging from simple instructions to sophisticated multi-step approaches (see Table 6). Of the 75 included studies, 46 (61%) included at least some prompt content, 19 (25%) described their approach only in general terms without reproducing prompts, and 10 (13%) reported no prompting details at all. Note that from published articles alone it is not possible to determine whether reported prompts represent the complete and verbatim instructions used. Zhou et al. [38] described a comprehensive prompt structure including role setting, task instruction, context description, input data, and output indicator. Mizumoto et al. [65] recommended structured prompts with clear definitions, examples, chain-of-thought reasoning, contrastive examples, and confidence scoring .

Several studies emphasized the importance of prompt engineering. López-Pérez et al. [53] demonstrated that iterative prompt refinement improved ChatGPT’s performance from Kappa values over 0.47 initially to over 0.79 after refinements. Fan et al. [39] showed that fine-tuned prompts allowed GPT-3.5-turbo to surpass GPT-4o performance. The most common approaches were iterative refinement (*n* = 49), involving multiple rounds of prompting to refine outputs. Other frequently used strategies included step-by-step instructions (*n* = 35, chain-of-thought prompting), and role assignment (*n* = 20, instructing the AI to act as a researcher). Few-shot learning (*n* = 10, providing examples) and zero-shot approaches (*n* = 10, no examples provided) were also employed.

Example prompts from the literature included:

- For thematic analysis [*role assignment*]: “Please act like a researcher with expertise in qualitative research and thematically analyze this transcript” [50].
- For coding [*structured output format*]: “The following codes were obtained via coding of a transcript. Please identify the overarching themes and subthemes as is done in thematic analysis. These themes should have as little overlap as possible, and will be used in a scientific paper focused on experiences of living with diabetes. Use the following format: […]” [58].
- For grounded theory [*role assignment with methodological framing*]: “You are a scholar in social sciences, skilled in using interview data and grounded theory […]” [26].
- For content analysis [*step-by-step instruction*]: “Please do a qualitative content analysis according to Mayring with the following interview excerpt” [74].

### Ethical considerations and human in the loop

Nearly all studies (95%) discussed ethical considerations related to LLM use. Common concerns included the potential for hallucinations, where models generate plausible but incorrect information (see Table 7). Balt et al. [88] specifically noted instances of “unsolicited elaboration and hallucination” in LLM summaries. Several studies highlighted cultural and linguistic limitations, with Sakaguchi et al. [50] finding that ChatGPT-4 struggled with themes requiring deeper cultural and emotional interpretation in Japanese contexts .

Data privacy emerged as a major concern, particularly for healthcare research. Mathis et al. [89] specifically addressed this by using locally-hosted open-source LLMs to avoid cloud-based privacy issues when processing protected health information. Qiao et al. [57] noted that privacy concerns must be acknowledged when using commercial tools for sensitive interview data.

Several studies advocated for collaborative models where LLMs enhance rather than replace human analysis. Balt et al. [88] recommended “a collaborative model, whereby the LLM’s deductive coding is complemented by review, inductive coding and further interpretation by a researcher”. Koželj et al. [40] employed a hybrid approach where human researchers established core concepts and LLMs assisted in recognizing patterns and generalizing themes .

The level of human involvement varied by research phase. Stage et al. [22] used ChatGPT for initial coding but required human researchers to perform later stages of thematic analysis and cross-check LLM outputs. Kondo et al. [24] found that “to achieve deeper analysis, it is essential to supplement the research context with human input” .

### Application domains and performance heterogeneity

LLM applications in qualitative research spanned diverse domains with healthcare emerging as the most prominent field. Studies analyzed patient interviews, healthcare provider perspectives, and processed large volumes of health-related social media data, such as tweets for epidemic indicators [85] and analysis of tweets about mask-wearing during COVID-19 [91]. Educational applications included analyzing student course evaluations and faculty assessments, with Brondani et al. [35] finding that instructors could differentiate ChatGPT-generated reflections from student work 85% of the time. Social science applications included Borgström et al.‘s [45] analysis of 45 years of Swedish parliamentary debates and Lee et al.‘s [27] examination of factors influencing Australians’ climate change opinions .

The substantial variation in reported agreement between LLM and human coders across these domains (from 36% to 99% agreement with human coders) reflects several interconnected factors. Task complexity proved critical, with straightforward classification achieving higher agreement than interpretive or culturally-nuanced analysis. Sakaguchi et al. [50] found ChatGPT-4 achieved over 80% agreement for descriptive themes like “personal experience” but only approximately 30% for themes requiring “deeper cultural and emotional interpretation” such as “fate”. Prompt engineering quality substantially influenced outcomes, as López-Pérez et al. [53] demonstrated refined prompts improved Kappa values from 0.47 to over 0.79. Finally, studies employing rigorous validation with multiple evaluation methods, independent human coders, and comprehensive codebooks provided more conservative estimates, potentially reflecting more accurate assessments of LLM performance.

## Discussion

### Principal findings

This scoping review of 75 studies provides an overview of the emerging use of LLM applications in qualitative research. The identified body of 75 studies published between January 2020 and May 2025 indicates that LLMs are now being applied across a range of qualitative research workflows. However, this review did not formally assess changes over time, and significant gaps in reporting, methodological standardization, and critical engagement persist. Key model parameters such as temperature and top_p govern the stochasticity and variability of model outputs, while context length determines how much input data the model can process. Without reporting these parameters, identical prompts and datasets may yield substantially different results, making it impossible to reproduce findings or assess their robustness. Moreover, incomplete reporting limits comparability across studies and constrain critical evaluation of methodological quality.

Our review demonstrates that LLMs are being applied across the full spectrum of qualitative research stages, from developing interview materials and transcribing data to supporting coding and theme development, summarizing datasets, and drafting analytic texts. While many studies in our review reported using LLMs for coding tasks, a closer examination reveals that most applications remain at the level of descriptive or deductive coding with predefined codebooks. This is consistent with recent empirical work showing that ChatGPT performs well for first-level open and descriptive coding but struggles with the interpretive depth required for second-level coding and thematic synthesis [26]. Schroeder et al. [14] found in interviews with 20 qualitative researchers and students that participants consistently emphasized that while LLMs can surface useful complementary insights, keeping meaning-making as a human-centered project is critical. This aligns with our finding that the majority of included studies maintained human oversight and positioned LLMs as assistive tools rather than autonomous analysts.

### Reporting practices and reproducibility

One of the most striking findings of this review is the extent of incomplete technical reporting. While nearly all studies specified model versions, only 13 reported temperature settings, 12 documented context length, and merely 4 provided top_p values. Similarly, while 46 studies provided complete or partial prompts, 10 reported no prompting details at all.

However, the concept of reproducibility requires careful consideration in the context of qualitative research. While reproducibility is a central goal in quantitative and computational research, qualitative approaches typically emphasize transparency, reflexivity, and transferability rather than replication of findings. In this context, the need for detailed reporting of LLM-related technical parameters (e.g., model version, temperature, prompting strategies) should be understood as supporting the reproducibility of computational processes, not the replication of qualitative interpretations. Accordingly, reporting practices for LLM-assisted qualitative research must balance technical transparency with the epistemological foundations of qualitative inquiry.

The recently published COREQ + LLM protocol [20] represents a direct response to this gap. Developed as an extension of the established COREQ checklist, COREQ + LLM aims to provide specific guidance for documenting LLM integration in qualitative research workflows, covering model specifications, configuration details, parameter settings, prompting strategies, and validation approaches. The findings of our scoping review directly informed the development of these reporting guidelines and underscore their urgency: without standardized reporting, the field risks producing a growing body of LLM-assisted qualitative research that cannot be evaluated, replicated, or meaningfully compared.

### Performance and limitations of LLMs

Performance metrics across the included studies varied substantially, with agreement rates between LLM and human coders ranging from 36% to 99%. This wide variability reflects differences in task complexity, prompt engineering quality, data characteristics, and model selection. High-quality prompts typically include detailed instructions (e.g., specifying coding procedures or analytical frameworks), constraints (e.g., minimizing overlap between themes), and structured output requirements, whereas vague or underspecified prompts may lead to inconsistent or superficial results. Our findings indicate that LLM applications are primarily concentrated in structured, descriptive tasks, whereas their use for more interpretive forms of qualitative analysis remains limited. As a scoping review, our study does not evaluate the effectiveness of LLMs across tasks but rather maps current applications and reporting practices. However, several empirical studies suggest that while LLMs perform well in more structured coding tasks, they face challenges in capturing deeper contextual and interpretive aspects of qualitative analysis.

Recent empirical work further illustrates this pattern. Yue et al. [26] reported strong consistency between ChatGPT 4-Turbo and manual coding at the sentence level for grounded theory, while also noting limitations in capturing depth, contextual connections, and nuanced code organization. Similarly, Liu et al. [94] found that GPT-4 can code a broad range of constructs, but that no single prompting method consistently outperformed others, with performance depending on the clarity, concreteness, and specificity of the construct being coded. Taken together, these studies suggest that the widespread narrative of LLMs as general-purpose qualitative analysis tools may require qualification. However, as this scoping review did not assess effectiveness, these observations reflect reported findings in the literature rather than conclusions derived from a systematic evaluation within this review.

### Implications for research and practice

Several implications emerge from this review. First, the development and adoption of LLM-specific reporting guidelines such as COREQ + LLM is an urgent priority. Our findings provide empirical evidence for what these guidelines must address: model version and provider, parameter settings (temperature, top_p, context length), complete prompt documentation, validation procedures, and reflexive documentation of how LLM outputs were integrated into the analysis. While reporting guidelines such as COREQ have been widely adopted to improve transparency and completeness in qualitative research reporting, their use has also been subject to ongoing debate within qualitative research communities. Critics have raised concerns that such guidelines may encourage standardized reporting practices that risk oversimplifying the inherently interpretive, context-dependent, and reflexive nature of qualitative research. There is also concern that checklist-based approaches may lead to superficial compliance (“box-ticking”) rather than meaningful engagement with methodological rigor. In addition, COREQ was originally developed to guide the reporting of interview- and focus group-based studies and therefore reflects only a subset of qualitative research methods. This is a relevant limitation, particularly given that LLMs are increasingly applied across a broader range of qualitative approaches. COREQ has also not been updated since its publication in 2007 and has received critique regarding its scope and applicability. Despite these limitations, COREQ remains one of the most widely used and recognized reporting frameworks in qualitative research and is familiar to both researchers and journal editors. For this reason, it provides a pragmatic starting point for developing an extension addressing LLM use. In this context, the proposed COREQ + LLM extension should not be understood as a prescriptive framework that constrains qualitative methodologies. Rather, it is intended as a tool to enhance transparency and reflexivity in the reporting of LLM integration, addressing an underreported methodological dimension while remaining compatible with diverse qualitative approaches.

Second, comparative methodological studies are needed that systematically examine different models, prompting strategies, and validation approaches across diverse qualitative tasks and data types. The reliance on OpenAI GPT models (93%) represents both a market reality and a critical gap in tool-knowledge among researchers. Studies comparing open-source alternatives (e.g., LLaMA, Mistral) with proprietary models would strengthen the evidence base and reduce vendor dependency.

Third, investigation of LLM applications in non-English and multilingual contexts is essential. Fourth, longitudinal research examining the effects of sustained LLM use on researcher skill development, analytical practices, and the evolution of qualitative methodologies will be important for understanding the long-term implications of this technological integration.

A critical gap identified through this review concerns the lack of robust evidence regarding the effectiveness of LLMs in qualitative research. While numerous studies report the use of LLMs for coding, theme development, and analysis support, very few provide systematic evaluations comparing LLM-generated outputs with high-quality human qualitative analysis.

Addressing this gap will require carefully designed empirical studies that directly compare LLM-assisted and human-led analyses. Such studies should involve experienced qualitative researchers, standardized datasets, and blinded evaluation procedures to assess the quality, depth, and interpretive validity of the analyses. Without this evidence base, claims regarding the utility or superiority of LLMs in qualitative research remain premature.

### Limitations of the review

Several limitations should be acknowledged. First, the search was limited to peer-reviewed journal articles published in English between January 2020 and May 2025, potentially excluding relevant work in other languages, conference proceedings, preprints, or grey literature where methodological innovations may first appear. The restriction to English-language publications may also introduce a language bias and limits the representation of non-English qualitative research traditions. Second, the geographic concentration of included studies limits generalizability and raises questions about applicability in diverse global research contexts. Third, consistent with scoping review methodology, no formal quality assessment was conducted, treating all studies equally regardless of methodological rigor. Fourth, the rapid evolution of LLM technology means that findings regarding specific models and their capabilities may quickly become dated. Fifth, our review focused exclusively on LLMs and did not include other AI-based tools (e.g., BERT-based classifiers, traditional NLP approaches) that may be used in qualitative research contexts, limiting the scope of our technological assessment. Given the rapid pace of development in LLM technologies and related research, the time lag between the literature search and manuscript submission represents an additional limitation. As a result, more recent studies and developments may not be fully captured, and the findings should be interpreted as reflecting the state of the evidence at the time of the search.

## Conclusion

This scoping review demonstrates that LLMs are being explored and applied across qualitative research workflows. However, the aims of the included studies indicate that many applications remain exploratory or evaluative in nature, rather than reflecting established or routine adoption. Additional technical reporting remains highly inconsistent: critical parameters such as temperature, context length, and exact prompts are reported in a minority of studies, directly undermining reproducibility and comparability. Given these findings, the development and adoption of dedicated reporting guidelines, such as the COREQ + LLM extension, is urgently needed to ensure that LLM-assisted qualitative research meets the standards of transparency, rigor, and interpretive depth that the field demands.

## Data Availability

No datasets were generated or analysed during the current study.
